# Telehealth consultations in general practice during a pandemic lockdown: survey and interviews on patient experiences and preferences

**DOI:** 10.1186/s12875-020-01336-1

**Published:** 2020-12-13

**Authors:** Fiona Imlach, Eileen McKinlay, Lesley Middleton, Jonathan Kennedy, Megan Pledger, Lynne Russell, Marianna Churchward, Jacqueline Cumming, Karen McBride-Henry

**Affiliations:** 1 Health Services Research Centre, Victoria University of Wellington, Old Government Building, 55 Lambton Quay, Wellington, 6011 New Zealand; 2 Department of Primary Health Care and General Practice, University of Otago Wellington, 23 Mein Street, Newtown, Wellington, 6242 New Zealand

**Keywords:** Telemedicine, Delivery of health care, Primary health care, Pandemic, Patient-centred care, Patient experience

## Abstract

**Background:**

During the first COVID-19 pandemic ‘lockdown’ in Aotearoa/New Zealand (March–May 2020, in which strict ‘stay at home’ measures were introduced), general practices were advised to use telephone and video consultations (telehealth) wherever possible instead of the usual in-person visits. This was a sudden change for most practices and patients. This research aimed to explore how patients accessed general practice during lockdown and evaluate their experiences with telehealth, to inform how telehealth could be most effectively used in the future.

**Methods:**

Using a mixed-method approach, we undertook an online survey and in-depth interviews with adults (> 18 years) who had contact with practices during lockdown, recruited through social media and email lists. We present descriptive statistics from the survey data (*n* = 1010) and qualitative analysis of interview data (*n* = 38) and open-ended survey questions, using a framework of access to health care, from the patient’s perspective.

**Results:**

In general, patients reported high satisfaction with telehealth in general practice during lockdown. Telehealth was convenient and allowed patients to safely access health care without having to weigh-up the fear of COVID-19 infection against the need to be seen. Telehealth worked best for routine and familiar health issues and when rapport was established between patients and clinicians. This was easier with a pre-existing clinical relationship, but not impossible without one. Telehealth was less suitable when a physical examination was needed, when the diagnosis was unknown or for patients who had a strong preference to be seen in-person.

**Conclusions:**

Even in this disruptive lockdown period, that prompted an unexpected and rapid implementation of telehealth services in general practices, most patients had positive experiences with telehealth. In the future, patients want the choice of consultation type to match their needs, circumstances, and preferences. Technological issues and funding barriers may need to be addressed, and clear communication for both patients and clinicians is needed about key aspects of telehealth (e.g. cost, appropriateness, privacy). Maintaining telehealth as an option post-lockdown has the potential to increase timely and safe access to primary health care for many patients.

**Supplementary Information:**

The online version contains supplementary material available at 10.1186/s12875-020-01336-1.

## Background

The first case of the new coronavirus COVID-19 was recorded in Aotearoa/New Zealand on 28 February 2020, nearly two weeks before a global pandemic was declared. In response, the Government introduced a four-level alert system that outlined actions to be taken to contain COVID-19 at different levels of risk [1]. On 23 March, Aotearoa/New Zealand moved into level 3, then to level 4 from 25 March to 27 April and back to level 3 until 13 May. Levels 3 and 4 were known as ‘lockdown’, in which all but essential workers stayed at home, physical distancing was required, and travel was restricted, including the closing of borders to all non-residents. During this first COVID-19 lockdown period, primary care services were advised to use ‘virtual’ consultations wherever possible, which in practice meant that every patient was expected to have a telephone or video triage consultation to judge the requirement to be seen in-person [2]. Reducing in-person consultations was intended to minimise the potential spread of COVID-19 through waiting rooms, particularly to patients at higher risk of infection, and to protect health professionals from the virus [2].

Before lockdown, most primary care in Aotearoa/New Zealand was delivered in-person, usually within a general practice (see The use of telehealth, defined as ‘remote delivery of health care services using information and communication technology’ [3] includes video conferencing, telephone, text, email and online patient portals. This had been slowly increasing within general practice but in an ad-hoc way. For example, around two thirds of practices were offering an online patient portal as of September 2019 but only 20% of patients at these practices were registered to use it [4]. Although some practices had implemented telephone triage and telephone consultations in recent years, [5] this had not yet become widespread or routine. According to the pre-COVID-19 literature, barriers to more widespread adoption of telehealth included resistance or inertia from health systems and health professionals (who are often more cautious than patients) and technological impediments. Specific barriers identified were problems with network/internet and equipment reliability, interconnectivity and privacy/hacking, lack of education for clinicians about use of appropriate technology, lack of supportive leadership and policies, and lack of funding [6–8]. The lockdown pushed general practices to adopt telehealth, irrespective of past reluctance from funders, professionals or patients, and bolstered by Government funding of $20 million to increase telehealth in response to COVID-19 [2].

Table 1 for a summary of the primary care system). The use of telehealth, defined as ‘remote delivery of health care services using information and communication technology’ [3] includes video conferencing, telephone, text, email and online patient portals. This had been slowly increasing within general practice but in an ad-hoc way. For example, around two thirds of practices were offering an online patient portal as of September 2019 but only 20% of patients at these practices were registered to use it [4]. Although some practices had implemented telephone triage and telephone consultations in recent years, [5] this had not yet become widespread or routine. According to the pre-COVID-19 literature, barriers to more widespread adoption of telehealth included resistance or inertia from health systems and health professionals (who are often more cautious than patients) and technological impediments. Specific barriers identified were problems with network/internet and equipment reliability, interconnectivity and privacy/hacking, lack of education for clinicians about use of appropriate technology, lack of supportive leadership and policies, and lack of funding [6–8]. The lockdown pushed general practices to adopt telehealth, irrespective of past reluctance from funders, professionals or patients, and bolstered by Government funding of $20 million to increase telehealth in response to COVID-19 [2].

**Table 1:** Aotearoa/New Zealand’s primary care system

Aotearoa/New Zealand has a strong first-contact primary care system where most general practices operate as small businesses. Currently, the Government pays a fixed amount per quarter for people enrolled in a general practice via weighted capitation payments, with higher payments made for population groups with higher health needs, for those on lower incomes, and for practices offering lower cost care. Standard consultations are typically free for children aged under 14 years. Patients aged 14 years and over are charged co-payments of varying amounts for services, which act as significant barriers to access, particularly for Māori, Pacific peoples and those on lower incomes. A typical charge for an adult is between $50 and $65 dollars; some practices in high deprivation areas are able to charge less. A separate accident compensation scheme subsidies accident-related care, with practices funded on a fee-for-service basis, with subsidies also adjusted for need, income and the offering of lower cost care [9].	

Access to health care is conceptualised by Levesque et al. as the abilities of individuals interacting with elements of service accessibility [10]. In this framework, there are five stages of accessing health care from the patient perspective, starting with the recognition of health care needs (ability to perceive). This prompts health care seeking (ability to seek), followed by actually getting (ability to reach) and utilising health care, which is affected by patients’ ability to pay and ability to engage, which will lead to improvements in health, provided the health care is effective. Research pre-COVID-19 showed that telehealth can improve access to health care for those in rural areas or with restricted ability to travel but those with poor internet connections (which, perversely, may be worse in rural areas), English as a second language or privacy concerns may be disadvantaged [11, 12]. Equitable access is foundational to good primary care [10, 13] and relates not only to patient characteristics but also to the quality and availability of services. In the context of lockdown, telehealth could potentially mitigate the expected negative impacts on access to general practice, by making services easier to reach, but it may also have had unexpected negative consequences for some.

This study focused on telephone and video consultations, henceforth described as ‘telehealth’ for the purpose of this research. The purpose of the study was to explore when, and for whom, telehealth could facilitate access to general practice, during lockdown and afterwards, and how telehealth affected the intersection between individual abilities and service accessibility. Lockdown provided a unique opportunity to draw on the experiences of those who had not previously had either the option or inclination to use telehealth. The study aimed to explore:

- What type of contact patients had with general practices during lockdown;
- Positive and negative patient experiences of telehealth during lockdown; and
- How patients would like to use telehealth in the future.

## Methods

We used a mixed-methods approach, collecting patient experiences through an anonymous online Qualtrics survey that included both closed and open-ended questions, supplemented by in-depth semi-structured interviews with a sample of survey respondents.

### Development and content

Survey questions asked whether or not respondents had contacted general practices, and if so, how and for whom. Those who had sought care were asked questions about their experiences of telehealth, including open-ended questions on what respondents liked and did not like about the consultations (survey questionnaire available in ‘Additional file 1’). These, along with responses to a general question ‘anything else you would like to tell us about your experiences of health care in the community during the pandemic’ were included in the qualitative data analysis. Respondents were also asked about their awareness of telehealth services in the past and what services they would like to use in the future. We included standard questions about the quality of services from the Primary Care Patient Experience Survey, [14] with minor modifications to make them applicable to telehealth. Demographic questions included age, gender, ethnicity, location, health status and presence of chronic conditions or disability. As a part of the in-depth interviews, respondents were asked to elaborate about their experiences of accessing general practice, including telehealth, during lockdown, and whether and in what circumstances they expected to use telehealth when lockdown was over. Given the constraints of lockdown, the questionnaire and interview schedule were informally reviewed by external experts, and pilot testing was conducted with household contacts of the research team. The qualitative schedule built on what had been asked in the survey and included an in-depth exploration of how people were managing lockdown with restricted movements and limited household contacts and wider ideas about wellbeing in a pandemic. It also explored topics of seeking health care and managing health conditions.

### Recruitment and data collection

#### Online survey

Recruitment for the online survey was through snowballing using digital media, as in other surveys during the pandemic [15, 16]. Advertisements for the survey were posted on personal and professional social media platforms, including Facebook, Twitter, Neighbourly (New Zealand-specific neighbourhood site) distributed and promoted through university channels and organisations such as Health Navigator, Healthline NZ, Primary Health Organisation quality improvement network, Diabetes NZ, Asian Health Network, Evolve Wellington Youth Service, and MenzShed NZ.

Respondents in the online survey were adults (18 years and older) who needed to contact general practices during the lockdown period. The survey was online from 20 April to 13 May 2020 and took a median of 12 min to complete. There were 1190 connections to the survey with 1010 respondents included in the final analysis. A requirement of the ethics committee was that no respondent should be obliged to answer any question. This meant that questions could have missing answers or that respondents could leave the survey without completing it; those with fewer than 20 pieces of information were excluded on this basis (*n* = 180).

#### Interviews

Survey respondents could provide their contact details (collected separately and disconnected from survey responses) if they were willing to be interviewed. From the 436 respondents who gave their details, we randomly selected 75, stratified by gender, to invite by email to a telephone or video (Zoom) interview. The email invitation outlined the purpose of the research and what an interview would involve. From these invitations, 41 agreed and 38 completed the interview within the timeframe available (the remainder did not respond). All interviewees provided oral or written consent prior to audio-recorded interviews being conducted via Zoom or telephone (on average 33 min). Interviews were transcribed and checked for accuracy. Interviewees were given the opportunity to review and amend transcripts. The research team agreed to complete the in-depth interviews once distinct commonalities were emerging from the participants.

### Data analysis

#### Quantitative survey data

As the survey was based on a self-selected convenience sample, only descriptive statistics are presented. Missing values were treated as completely missing at random, assuming that survey respondents who did not provide answers were similar to respondents who did.

#### Qualitative survey and interview data

We used thematic analysis and a mixture of deductive and inductive coding to analyse the qualitative data, [17, 18] which included the 38 interview transcripts and responses to open-ended survey questions. Five hundred and five survey respondents answered questions on what they liked and did not like about telephone consultations; 41 answered what they liked and did not like about video consultations; and 475 had more to tell us about health care during the pandemic. NVivo (QSR International Pty Ltd. Version 12, 2018) was used to manage the datasets.

We used Levesque’s conceptualisation of access to health care [10] as a framework for analysis, testing a preliminary coding scheme against the interview data and making revisions as additional themes emerged. In this analysis, our survey respondents had already recognised a health need, and some had sought health care. Hence, we focused on themes related to the ability to reach, ability to pay and ability to engage. This provided background to understanding why there were different preferences for telehealth.

Themes from the interviews were checked against the analysis of the open-ended questions for consistency and to confirm we had not missed any major findings. The coders used defined nodes for coding and the team met regularly to discuss the data, coding framework and emerging themes; final decisions about themes were made by consensus. Quotes are inserted verbatim, with identifiers including age range, gender and whether from survey or interview (S or I).

## Results

### Sample characteristics

#### Survey sample

The survey respondents were mostly female, with more Europeans and fewer older adults than expected (Table 2). The highest proportion of responses came from localities related to the research team’s institutions (Wellington and Otago, with an associated city), due to the snow-balling recruitment strategy, which started survey dissemination through the researchers’ networks. We compared the survey sample with results from the nationally representative New Zealand Health Survey (see ‘Additional file 2’). A large proportion of survey respondents reported having a chronic health condition, which may have been due to the broad definition in the question (‘any health conditions that mean you have to contact a GP clinic/health centre on an on-going basis’). Conversely, fewer respondents reported having a disability than would be expected from other estimates [19], which may be because we did not ask about specific disabilities, such as hearing or sight impairment, but asked for a subjective assessment (‘do you think of yourself as disabled?’).

**Table 2:** Demographics of survey respondents and interviewees

Characteristic	Survey respondents (*n* = 1010) %	Interviewees (*n* = 38) %
**Age group (years)**		
18–34	22.4	18
35–44	20.4	16
45–54	25.0	32
55–64	17.5	8
65+	14.7	26
**Gender**		
Female	84.5	63
Male	14.2	37
Other^a,b^	1.3	–
**Prioritised ethnicity (in priority order)**		
Māori	10.2	16
Pacific peoples	1.8	8
Asian	3.4	11
New Zealand European/Other	84.5	66
**Current work status**		
In paid employment as before COVID-19	58.9	58
In paid employment with reduced pay due to COVID-19	10.9	8
In paid employment but not being paid due to COVID-19	2.6	–
Unemployed and looking for a job	3.1	–
Not in paid employment and not looking for a job	24.3	34
**Struggle to pay for basic living costs**		
Agree/Strongly agree	7.5	
Neither	10.7	
Disagree/Strongly disagree	81.9	
**Self-rated health**		
Excellent	12.1	
Very good	38.7	
Good	32.4	
Fair	13.9	
Poor	3.0	
**Presence of one or more long term health conditions**	60.9	
**Presence of disability**	12.6	
**Grouped District Health Board (DHB) region**^b^		
Northern region	20.7	18
Midland region	11.9	8
Central region	44.1	53
South Island	23.4	21

^a^ Those who answered “gender diverse” or “prefer not to say” were grouped together because of small numbers; ^b^Northern region = Northland, Waitematā, Auckland and Counties Manukau DHBs; Midland region = Waikato, Bay of Plenty, Tairāwhiti, Lakes, Taranaki DHBs; Central region = Whanganui, Hawke’s Bay, MidCentral, Wairarapa, Hutt, Capital and Coast DHBs; South Island = Nelson-Marlborough, West Coast, Canterbury, South Canterbury, Southern DHBs

Only 3.1% of survey respondents were unemployed, compared with the national unemployment rate of 4.2% in the period before lock-down [20]. However, more respondents (7.5%) reported struggling to pay for basic living costs, compared with between 4 and 6% from a weekly survey at the same time, which asked the same question [21].

#### Interview sample

Since the interviewees were recruited from the survey, they were also more likely to be from the Wellington and Otago regions (Table 2). In contrast to the survey respondents, however, interviewees were more likely to be older and 34% were not employed or seeking work. They may have had more time and willingness to undertake an interview. We stratified potential interviewees by gender using the names they supplied since the survey respondents were disproportionately female. We were unable to target any other demographic as the contact details for interviewees could not be linked back to survey data.

### Survey quantitative results

#### Contacts with general practice

Of the 1010 survey respondents, 86% (866) had contacted general practice during lockdown (others may have wanted to but did not, as the survey also asked about reasons for delaying seeking health care). For those who made contact, the top methods (multiple options were allowed) were by telephone (85%), through an online patient portal or website (30%), by visiting the clinic (26%) or by email (15%). Almost all of those who used text and instant messaging (32 responses) also used one of the other methods. Contact method did not vary by age or gender. Those with one or more long-term health conditions reported a higher use of online patient portals/website, email and visiting the clinic. Just over half (54%) of those who made contact did so on more than one occasion during lockdown (up until the time they completed the survey).

The most common reasons for contacting general practices (multiple reasons allowed) were for routine or non-urgent issues (42%) including vaccinations and medical certificates; repeat prescriptions (41%); an urgent or persistent issue (39%) such as injury, infection and pain; or chronic health conditions (25%). Known or suspected COVID-19 infection was a cause for contact for 10% of respondents.

#### Experience and satisfaction with telehealth

Survey respondents who had contacted general practices were asked in more detail about their experiences of telehealth and in-person consultations during lockdown. Of those who had any kind of consultation, 61% (528) had a telephone consultation, 5% [22] had a video consultation and 39% (337) had an in-person visit. Respondents who had telephone consultations had very similar sociodemographic characteristics to those who had in-person visits. Video consultations were too few to compare by patient characteristics.

Most respondents accessed telehealth for themselves, but 14% were for a child or another person (e.g. an older family member). Most consultations were with a doctor (84%). Only 17% had experience of telehealth before the lockdown. Consultations commonly cost the same as a regular visit (43% for telephone and 46% of video consultations), with only 14% of telephone consultations charged at a lower rate. Around 22% of telehealth consultations were free (e.g. for accidents or children aged under 14 years, which are covered by specific government funding). The cost was not known or reported for 18% of telephone and 26% of video consultations. From the qualitative data, this may have been because patients were not told/did not ask about the cost before the consultation, they could not remember, or they had not yet received an invoice.

Overall satisfaction with telehealth was high, at 91% for video and 86% for telephone consultations, but was slightly lower than in-person visits (92%) (Table 3). Some of the difference in satisfaction between telehealth and in-person visits related to how well practice staff were reported to ‘spend enough time with you’ and ‘listen to what you had to say’ (Table 4). In addition, some respondents expressed concern about not being seen, with 29% of respondents who had telephone consultations and 36% of respondents who had video consultations being moderately, very, or extremely, concerned that they could not be physically examined.

**Table 3:** Type of consult and satisfaction by survey respondent characteristics

Characteristic	Satisfied^a^ with telephone consultation (*n* = 454) %	Satisfied^a^ with in person visit (*n* = 309) %
**Overall**	**86**	**92**
**Age group (years)**		
18–34	88	93
35–44	80	86
45–54	86	93
55–64	89	89
65+	89	96
**Gender**		
Female	87	91
Male	82	92
Other^b^	57^c^	83^c^
**Prioritised ethnicity (in priority order)**		
Māori	82	90
Pacific peoples	86^c^	80^c^
Asian	60^c^	80^c^
New Zealand European/Other	88	92
**DHB region**		
Northern region	82	90
Midlands region	75	82
Central region	88	90
South Island	91	99
**Current work status**		
In paid employment as before COVID-19	88	92
In paid employment with reduced pay due to COVID-19	82	88
In paid employment but not being paid due to COVID-19	90^c^	100^c^
Unemployed and looking for a job	69^c^	75^c^
Not in paid employment and not looking	86	82
**Long term health condition(s)**		
Yes	87	91
No	84	91
**Disability**		
Yes	92	89
No	85	92
**Self-rated health**		
Excellent	95	94
Very good	87	95
Good	86	90
Fair	83	91
Poor	61	58^c^
**Struggle to pay for basic living costs over past seven days**		
Strongly agree or agree	86	88
Neither agree nor disagree	69	90
Strongly disagree or disagree	89	92

^a^Group Satisfied = responses of “Very satisfied” or “Satisfied”; ^b^Those who answered “gender diverse” or “prefer not to say” were grouped together because of small numbers; ^c^Based on < 10 responses”

**Table 4:** Quality measures of interaction with the health professional by consultation type

Quality measure	Consult type		
	Telephone (***n*** = 528) %	Video (***n*** = 46) %	In-person (***n*** = 337) %
**Did your doctor or nurse …**^a^			
listen to what you had to say	98	98	97
*- Yes, but not as well as in-person*	*20*	–	n/a
spend enough time with you	95	98	95
*- Yes, but not as well as in-person*	*23*	–	n/a
treat you with kindness	98	98	97
*- Yes, but not as well as in-person*	*10*	–	n/a
explain things to you in a way that was easy to understand	97	98	98
*- Yes, but not as well as in-person*	*12*	–	n/a

^a^Combined responses for ‘Yes, just as well as in-person’ and ‘Yes, but not as well as in-person’ (for telephone/video consultation) and ‘Yes’ (for In-person consultation). Responses for ‘Yes, but not as well as in-person’ not provided for video consultations due to small numbers

#### Future interest in telehealth

Survey respondents who had contact with general practice during lockdown were asked whether they were aware of telehealth methods before lockdown: 12% were aware of video consultations and 48% of telephone consultations. All respondents were asked what telehealth services they would like in the future; 80% wanted telephone and 69% wanted video consultations, indicating a willingness to retain these approaches to delivering health care. These future preferences did not vary markedly by age, gender, ethnicity, presence of health conditions or disability.

### Qualitative results

Themes from the qualitative data (interviews and survey open-ended questions) related to three of Levesque et al’s ‘patient ability’ dimensions of the access to health care framework [10]: convenience (ability to reach), views on value (ability to pay) and relationships, technology and the need to be seen (ability to engage). These themes were also consistent with research on how telehealth works to support self-management: relationships, fit (convenience and technology) and visibility (the need to be seen) [23].

In general, and consistent with the high satisfaction ratings from the survey, positive feedback on telehealth was more common than negative feedback. However, respondents reported mixed experiences for all types of consultations (telehealth and in-person). The themes from the interviews are discussed below, in order of how frequently they arose from the patients’ feedback.

#### Convenience

Many respondents mentioned the convenience of telehealth consultations, in terms of saving time and money, and reducing stress, travel, employment disruption and exposure to infection (with COVID-19 and other pathogens). Respondents highlighted the ease of having consultations that fitted around their day, with the “hassle of lots of waiting time and the usual transport issues (including parking)”(S: M, 55–64) being avoided and “all my health needs [being] met whilst in the comfort of my home”(S: M, 35–44). The convenience of telehealth mostly outweighed concerns about not being seen or examined when the health issue was relatively routine or familiar and when there was an existing trusted relationship with the health provider.

It did not bother me at all that I couldn't see the doctor - she knows me and my health background so this was not a barrier at all. (S: F, 45–54)

Paradoxically, lockdown itself led to better access and more convenient care for some survey respondents due to an overall decrease in demand, so that many practices had more appointments available, could respond more promptly and had more time to spend with patients.

So, BC [Before Corona] the most recent wait was 6 weeks, but on average it was at least 2-3 weeks, that’s when it was pretty good. During Corona... I rang and I had a phone appointment the next day. (I: F, 45–54)

Conversely, some found care more difficult to access, particularly if contacting practices by telephone and at the start of lockdown when many practices deactivated their patient online portals and patients were required to phone rather than book appointments online.

The online portal was shut down at start of pandemic so the only way to access GP appointment or repeat prescription was to ring. … I found it aggravating having to ring for a repeat prescription and wait ages. (S: F, 55–64)

Patients noticed that during lockdown they were not required to have an in-person visit for routine issues like repeat prescriptions, and that such visits pre-lockdown were not only inconvenient but unnecessary. This meant the convenience of well-functioning telehealth could improve access to primary care, including for groups that have previously not engaged as much with health services.

I didn’t have to go and take time off work to go to the general practice... So that’s where the convenience comes in; it makes the consult more efficient and perhaps it would encourage me, as a male, to actually go. (I: M, 65+)

#### Need to be seen in-person

Physical examination and observations were considered essential for some health concerns (e.g. IUD removal or a prostate check) and to give peace-of-mind and confidence. As a result, telephone and video consultations raised concerns for some participants about not being seen or adequately examined. Respondents indicated that telehealth worked especially well for non-urgent issues that did not require physical assessment, conditions that were familiar or where the patient knew what was wrong. Acute, new issues or more complex issues could be more difficult, especially if physical signs had to be elicited over the phone.

My condition was something that needed to be seen, even though I had photos sent and I described and showed it on video. I think it would have been a lot better if she could have seen it and felt it. (I: F, 25–34)

Within the lockdown context, respondents recognised the tension between the need to be seen and the benefits from keeping them and others safe from infection through physical distancing. Respondents adapted to the lockdown telehealth imperative by using ‘workarounds’ such as sending photos, emailing home blood pressure readings, and moving between phone and video consultations for visual assessment, in which case, video consultations had advantages over the telephone. On occasion, pragmatic, but less-than-ideal management of issues occurred.

It was difficult to get a diagnosis for an illness I had due to being unable to be seen in person or get a sample tested … I had to take antibiotics "just in case" it was a bacterial infection even though this was undetermined. (S: F, 25–34)

#### Relationships

Successful telehealth consultations required mutual trust between patient and clinician, which was easier when there was a pre-existing relationship. Consultations with clinicians who knew their medical history was reassuring and, for many, the telehealth experience was just like a regular consultation. For those who highly valued continuity of care, connecting with a known, trusted doctor was more important than having an in-person visit.

I've got a good relationship with my doctor too, and I think she trusts my description of what might be going on. This made the telephone consultation really easy. (S: F, 45–54)

I would prefer to do phone or video [consultation] with my own doctor than see a different doctor. (I: F, 45–54)

A pre-existing relationship, however, was not sufficient for a successful consultation when clinicians did not pay attention to establishing rapport within the telehealth environment. On the other hand, even when the respondent did not have a pre-existing relationship with the clinician, consultations could still be successful if the clinician created rapport.

It [phone consultation] was quite comfortable even though I’ve never done it before, and I didn’t know the doctor, but she was very kind very caring, and it came through in the call. (I: F, 45–54)

Some patients found telehealth less rushed, more focused and personal, even providing space to talk more freely than usual. Patients valued being reassured and having a calm, unhurried telehealth consultation in which they felt heard, with all their concerns addressed. Demonstrating active listening was even more important when visual cues were not available. For others, telehealth felt abrupt and impersonal, even if the clinician at the other end was known to the patient.

It’s kind of a bit more dismissive on the phone, you know … I just don’t feel comfortable. I just feel like it’s more human [in-person] than on the phone. (I: F, 35–44)

#### Technological barriers

Technological barriers to telehealth included connectivity problems including poor internet access or cellphone service, lack of phone credit or data, and patient or clinician lack of familiarity with online tools. Respondents felt that better use of video technology should have reduced the need for in-person visits, but this did not always eventuate, with reports of insufficient broadband speed or unstable internet connection, poor image resolution and poorly angled cameras. Poor sound quality could present a problem for anyone, but telephone consultations could be impossible for those who were hard of hearing.

I would rather face-to-face. Not telephone. My hearing is not good, partly deaf. (I: M, 65+)

The introduction of online payments was disconcerting for some, especially those in older age groups who were unused to online banking. Concerns were also raised at how some people could be excluded from accessing general practices by telehealth due to lack of support, resources or infrastructure. Respondents felt some level of support could be provided by the health service (e.g. advice and assistance in preparing for a video consultation), but suggested inadequate resources and infrastructure pointed to deeper societal inequities (e.g. poverty, differences in rural and urban access to technological services).

...some whānau [family] don’t have the finances for the technology needed to access online support or the know how to even navigate the internet. (S: F, 45–54)

...where they were [remote rural area], couldn’t get the internet and sometimes couldn’t get cell service. (I: F, 55–64)

Concerns about security and privacy were infrequent and mostly related to the fact that many telehealth consultations were taking place in a home environment, which may be unusually full of people, some of whom the patient might not want to overhear what was said.

Sometimes people are not comfortable discussing health issues from within their homes (lack of privacy, unsafe environment to discuss concerns etc.) (S: F, 18–24)

#### Views on value

Cost was not mentioned in survey responses as frequently as the previous themes. Payment for telehealth varied between general practices. Often patients were not advised about the charges or method of payment before consultations and clinicians themselves could be uncertain about the fee and how the payment would be arranged. Patients conveyed clear views on what was value for money, depending on the time spent with them and service provided. They were willing to pay the same fee as an in-person visit for telehealth, as long as it seemed commensurate with an in-person visit and met their health needs.

I got charged the same amount as normal … I got the same service as normal, so I guess it’s fine, but I guess the doctor did the same amount of work. (I: M, 45–54)

For some patients, it felt inappropriate for telehealth to be charged the same as an in-person visit when they could not be examined thoroughly and their issue was not resolved. Reservations also arose over whether short telehealth consultations should be charged the same as a lengthy in-person visit and whether a telehealth consultation for an issue that then required an in-person visit should be charged twice.

I was shocked I was charged $56.50 for a phone consult that lasted 10 minutes and did not include an examination of the affected area. (S: F, 45–54)

#### Patient preferences

Patient preferences for telehealth were also influenced by personal factors. Patients carefully judged when they were comfortable with telehealth and when they wanted to be seen in-person. Partly, this was due to the health issues they were presenting with. They weighed up considerations such as symptom severity, the likelihood of needing a physical examination and whether they could explain themselves more clearly in-person.

...if I was getting prescriptions from [doctor] this kind of format into the future would be absolutely fine. But if she needed to check my glands ... that becomes a little bit more difficult. (I: F, 45–54)

Individual preference for a type of interaction could override what might be judged appropriate based only on the health concern. Some patients wanted to be seen regardless of the concern, because touch, examination and social contact were more important to them than convenience. Others thought the option of telehealth was important because it could save time and money.

I hope that phone and video consultations remain an option long term as it is much quicker for routine things that don't require a physical examination. With travel and waiting time, I have to allow an hour for a GP appointment. The phone consult was done in 10 minutes. (S: F, 45–54)

Patients’s preferences for either telephone or video consultations were also highly individualised and context-dependent. For some, setting up video consultations was more stressful and difficult than telephone calls, which could be done from bed or wherever they happened to be at the time. Others relished the opportunity to connect digitally. Ultimately, patients wanted choice that was appropriately aligned with their needs and preferences.

The biggest thing for me I guess is as a patient or client to somehow know that there was still a choice around what way I want to connect with my GP. (I: M, 25–34)

## Discussion

### Summary

The majority of patients in this study responded positively to the abrupt switch to telehealth in general practice during the March–May 2020 lockdown period. This was despite the impact of the sudden disruption on systems that would have affected patient’s experiences, especially in the initial stages, and despite telehealth being involuntary in this context and previously uncommon (only 17% had experienced telehealth previously). Many patients valued the convenience and efficiency of telehealth and the ability to be ‘seen’ without risking exposure to infection. However, telehealth was not suitable for all people or for all issues. Some valued having a direct connection with their clinician (with the opportunity if needed for a physical examination) more highly than the convenience of telehealth, which may be particularly pertinent for areas like palliative care [24]. Others had a health condition that required a physical examination. Concerns about not being seen and telehealth consultations that felt rushed or uncaring meant that telehealth was not rated as highly as in-person visits on some aspects of quality of care. Despite these differences, most survey respondents, irrespective of age or other characteristics, were willing to try telehealth in the future.

### Comparison with existing research

Previous research on telehealth has confirmed that the use of telephone triage and consultations in general practice can reduce in-person visits, [25] with telehealth increasing access to health care and patients’ ability to self-manage chronic conditions [26] and significantly reducing patients’ visit times in outpatient clinics [27]. Other research has found patients choose telehealth because of its convenience, while in-person visits are chosen due to personal preference or technological barriers [28]. A survey of patients’ perceptions of telehealth in the United States of America during the COVID-19 pandemic found satisfaction was high both in new and previous users of telehealth but new users were more motivated to avoid waiting rooms and potential infection [16].

Research into telehealth before the COVID-19 pandemic mostly focused on the management or prevention of chronic conditions, and often in hospital outpatient settings [28–30]. In this context, telehealth (mostly video consultations) led to high patient satisfaction and equivalent health care outcomes, although it could not substitute for in-person care in all cases [31–33]. The current research supports these findings within the context of telehealth in general practice in lockdown.

Patients in this study thought carefully about when telehealth would or would not be suitable. Their assessments aligned with clinical opinion that remote consultations can be unsafe for rare, unknown or unstable conditions or when physical examination is needed and is most likely to be successful when patients are well-versed about their condition and communication with their clinician is effective [30]. Similarly, patients approved of telehealth for more routine issues, consistent with recent advice [29]. Other studies have also found that supportive clinical relationships and access to the internet and related technologies are needed for telehealth to work well, which correlates with the experiences in our survey [23, 34]. Lower quality ratings for telephone consultations also occurred in a UK study, associated with shorter consultation times and poorer information exchange [35].

### Strengths and limitations

The main limitation of this research is the sampling frame, which limits the generalisability of the results particularly to under-represented groups such as Pacific peoples and those without the access or ability to complete online surveys. Reporting of results for Māori respondents is planned for a separate publication. We did not have access to contact details for general practice users in Aotearoa/New Zealand and could not recruit in-person via practices because of the pandemic; thus, the online snow-ball recruitment approach was the most practicable option for obtaining rapid responses in a changing environment. A major justification for accepting this recruitment method was that the national Primary Care Patient Experience Survey in Aotearoa/New Zealand was not in the field during lockdown and although other surveys were assessing practices’ experiences of COVID-19, the patient perspective was at risk of being overlooked. This sampling frame also most likely contributed to the high proportion of female respondents. This is likely due to a variety of reasons, for example, women more likely to complete online surveys than men [36], more women than men attend primary care [37]. This survey also allowed people to respond on behalf of others (mothers much more likely to seek healthcare for children than fathers) [38]. Whether this has caused gender bias in the results is uncertain – some research has found that satisfaction with telehealth is higher in females [39, 40] so the level of satisfaction in this survey may be higher than in the general population. Interviewees were also a self-selecting group, likely those who were already more familiar and comfortable with telehealth methods of engagement. In addition, only those with online capability will have had the opportunity to complete the questionnaire; this would likely have biased the satisfaction upwards but we would expect the issues found in the qualitative results (especially technological barriers, the importance of relationships and choice) would also apply to those with limited technological ability.

Collection of patient experiences is essential for evaluation and service improvement, especially when changes to services delivery are novel and unexpected [41]. Also, the research finding that patients wanted choice and flexibility to engage with health services in a way that met their health needs could be reasonably expected to apply to populations beyond the study sample.

This research is a snapshot of a unique circumstance and the lockdown context could influence how patients perceived telehealth. For example, the high level of satisfaction with telehealth could be due to lower expectations of general practice during this time, especially when many believed that health services were less available. In fact, access to care improved for many patients who contacted practices during lockdown, because of lower demand, which also could have increased the positive feedback. Telehealth was appreciated because patients did not have to leave the house and risk infection but may be less attractive without the fear of COVID-19. Conversely, the lockdown context is widespread around the world and is likely to remain so to varying degrees in coming years. Therefore, even findings that are harder to generalise to circumstances post COVID-19 will have significant ongoing applicability.

A strength of this research was the mixed-method approach. We triangulated qualitative results through comparing responses from open-ended survey questions with interview responses and against the survey quantitative data. Qualitative results consistently confirmed survey responses but provided more nuanced insights. Although we interviewed 38 *individuals*, many of these interviewees also talked about the experiences of close family and friends, meaning that we heard about the experiences of many more than 38 people. This is both a strength and a weakness (because of the interpretative nature of second-hand accounts).

### Implications

After the March–May lockdown, general practices in Aotearoa/New Zealand reverted to seeing most patients in-person, although telehealth (particularly telephone consultations) were still offered by most practices [42]. A survey of practices in June 2020 revealed that only 27% judged that patients booked themselves appropriately into an in-person or telehealth consultation [43]. This contrasts with our findings, where most patients felt able to identify when telehealth was appropriate. More investigation is needed into why and when clinicians have different views on appropriate use of telehealth, and how much this is due to technical limitations, poor connectivity, lack of support or training and clinicians’ own skills, preferences, expectations and cultural differences.

Practical recommendations for improving the telehealth experience for patients relate primarily to clear and thoughtful communication. For example, in this research some respondents reported waiting hours for the expected telehealth consultation, which caused anxiety and was problematic for workers who had other video or phone meetings booked in the day. Practices should therefore notify patients of significant delays, in recognition that when patients are waiting remotely, they have no cues about when they will be ‘seen’. Other research also specifies the need for clarity around contact information, processes, and expectations, particularly communication about the time and purpose of appointments, who the appointment is with and what to do if technology fails or the call is missed [44]. Other procedural aspects to clarify are the cost and payment process for telehealth, whether the patient wants to be booked with their usual clinician or instead wants to take the next available appointment. In telehealth consultations themselves, privacy parameters can be established by clinicians and patients exchanging information about where they are (private or public space in home or practice) and who else is present. Active building of rapport is particularly important when the clinician has not met the patient previously, and in telephone consultations where the usual visual cues are missing.

Patient-centred general practices could also routinely ask about and record all patients’ preferences for phone or video consultations and offer telehealth in suitable circumstances. Guidance for both patients and practices on when telehealth is appropriate, how to weigh up the risks and benefits, and how to deliver telehealth safety is already available [30, 45, 46]. Proactive support for patients who lack technology capability or literacy can build proficiency and improve access to telehealth, [22] but dedicated resources are needed to promote this and ensure that no patient group misses out. More research is needed on whether telehealth can reduce inequalities, depending on how differential access to technology can be addressed.

There is an expectation that telehealth that can be provided more quickly and conveniently should also be cheaper for patients. However, this conflicts with business models of general practice that rely on co-payments, which can incentivise more expensive services (in-person rather than telehealth). More consideration is needed about how telehealth is funded, including set-up costs to practices for video consultations, level of co-payments, and connection costs to patients (e.g. phone credit and internet data).

## Conclusions

Past research has demonstrated the potential for telehealth (telephone and video) consultations to increase access to health care and provide services that are as good as in-person visits, but telehealth has yet to be fully embedded into general practice in Aotearoa/New Zealand. During the first lockdown, patients felt listened to during telehealth consultations, had a fair idea of what they felt was value-for-money and felt confident to judge when telehealth was appropriate and when they needed to be seen in-person. A particularly important characteristic of successful telehealth was the development of a mutually-trusting relationship between the clinician and the patient.​.

For most patients, telehealth can be as good, or even better than, in-person care, especially for those who face geographical and time barriers to access. For others, telehealth will not be as good as in-person visits, but it was important to respondents to have the choice and flexibility to engage with health services in the way that was most appropriate.

In this lockdown, disruption was an inadvertent instrument of innovation and change that had some positive impacts on health service delivery for those who accessed care during this time. The challenge is now to embed positive changes so that they become a normal part of general practice systems. Telehealth has been a critical part of controlling COVID-19, allowing people who need health care to be remotely assessed, protecting patients and health providers from unnecessary exposure to the virus. Looking ahead, the routine offering of telehealth could reduce indirect costs to patients, such as time and money lost in travel, missed work and waiting rooms. Thus, it has the potential to increase access to health care, [47] particularly if telehealth can be used to support patients’ self-management of routine or stable health issues, allowing more time for in-person visits for those with more complex needs [48]. Time will tell if the seeds of telehealth that were sown during lockdown will thrive as a part of health care delivery, as this research suggests that they should.

## Supplementary Information

**Additional file 1.** Online survey questionnaire.

**Additional file 2.** Survey data comparison.

## Data Availability

The datasets generated and analysed during the current study are not publicly available due to ethics committee approval requiring the restriction of data access to the research team as part of the data management conditions but may be available from the corresponding author on request and agreement of the ethics committee.

## Abbreviations

DHB: District Health Board, which is a regional organisation funded by the Ministry of Health with the responsibility for providing health services to populations within a defined geographical area
NZHS: New Zealand Health Survey
Whānau: Māori word for family or families
