# Supplementary material for: Telehealth consultations in general practice during a pandemic lockdown: survey and interviews on patient experiences and preferences
Source: BMC Fam Pract. 2020 Dec 13;21:269. doi: 10.1186/s12875-020-01336-1 (PMC7733693; doi:10.1186/s12875-020-01336-1)
Supplement: Supplementary file 1 — Additional file 1. Online survey questionnaire. [file 12875_2020_1336_MOESM1_ESM.docx]

# Health care in the community during the coronavirus pandemic

## Introduction

Thank you for considering taking part in this survey about health care in New Zealand during the COVID-19 pandemic. If you are not living in New Zealand, this survey will not be relevant to you.

**What is this survey about?**

On 23 March, New Zealand went to Alert Level 3 in response to the coronavirus pandemic, moving to Alert Level 4 on 25 March. At these Alert Levels, doctors and nurses in the community (in GP clinics, health or medical centres etc), have had to change the way they see and talk to patients.

We want to find out what changes you have experienced when contacting your doctor during this time. We are interested in what you think of these changes. If you wanted to get help for a health issue but put this off because of the pandemic, we also want to hear from you.

**How can you help?**

If you are 18 years or older and you have contacted (or wanted to contact) a GP clinic, health or medical centre since 23 March, we want to hear from you. The contact may have been by telephone, video, email, through a patient portal (e.g. Manage My Health) or face-to-face.

**Why do this survey?**

This survey will help us find out what changes to health care in the community are working well for people and whether these changes should carry on after the pandemic. We will provide the results to groups involved in health care services to help them decide what changes to make in the future. We will use the information from this survey in academic publications, presentations and seminars.

**What is involved?**

The survey will ask you about your contact with and experience of health services in the community. It will also ask about whether you put off seeking health care and what type of services you would like to see in the future. It will take up to 20 minutes to complete. By answering the survey, you are consenting for us to use your responses in this research.

**What happens to the information you give?**

Your responses are completely anonymous – nothing you say in the survey will be linked back to you. Once you submit the survey, it will be impossible to remove your answers. If you want to receive a copy of the research results, or be contacted by the researchers for further research, your personal contact information will be held in confidence and separately from the survey data. The survey data will be held securely on a server at Victoria University of Wellington for 5 years after publication of results.

**Thank you for taking our survey! Press the arrow button below to begin.**

**About the researchers**

We are a group of researchers from Victoria University of Wellington, the University of Otago and the University of Auckland. We do research on how well the health care system is working in New Zealand.

If you have any questions about this research, now or in the future, please contact the lead researcher:

Dr Fiona Imlach (Senior Researcher) from Victoria University of Wellington at Fiona.imlach@vuw.ac.nz or 022 563 6565

This research has been approved by the Human Ethics Committee at Victoria University of Wellington (ID: 0000028485). If you have any concerns about the ethical conduct of the research you can contact the Human Ethics Committee Convenor (Dr Judith Loveridge, email hec@vuw.ac.nz or telephone (04) 463 6028). The research is funded by the Health Research Council of New Zealand.

1. Have you contacted a Doctor’s or GP clinic, community health or medical centre, general

or family practice since 23 March 2020 (when Alert Level 3 started)? (The contact may have been by telephone, video, email, through a patient portal or face-to-face.)

- 1. Yes – for myself
  2. Yes – on behalf of someone else (e.g. a child or other family member)
  3. No

**If 1a or 1b, go to question 2. If 1c, go to question 48**

1. What is the name of the GP clinic/health centre you had contact with?

[if more than one, please note which one you had the most contact with. If you don't know or don't want to put down the name, can you tell us in what area of New Zealand it is.]

1. Was this GP clinic/health centre where you usually go?
   1. Yes
   2. No because I do not have a place that I usually go to
   3. No for some other reason (please specify)
2. How often have you contacted the GP clinic/health centre since 23 March 2020 (your best guess is fine)?
   1. Once
   2. Two or three times
   3. Four or five times
   4. More than five times
3. Since 23 March 2020, how have you contacted the GP clinic/health centre? [tick all that apply]
   1. By telephone
   2. By text
   3. By email
   4. Through an online patient portal (such as Manage My Health or MyIndici)
   5. By instant messaging
   6. By visiting the GP clinic/health centre
   7. Some other way (please specify)
4. Since 23 March 2020, what have you contacted the GP clinic/health centre about? [tick all that apply]
   1. A suspected or known coronavirus infection
   2. A repeat prescription
   3. An acute, urgent health issue (e.g. asthma attack, injury, other infection, pain)
   4. An ongoing, chronic health condition (e.g. diabetes, heart or lung problems, mental health condition)
   5. A routine non-urgent issue (e.g. flu vaccine, pregnancy check-up, child immunisation, contraception, screening)
   6. Something else (please specify)
5. Since 23 March 2020, have you had a **telephone consult** with the GP clinic/health centre?
   1. Yes
   2. No

**If 7a, go to question 8 if 7b go to question 20**

1. Who was the telephone consult with?
   1. A doctor
   2. A nurse
   3. Someone else (please specify)
2. Who was the telephone consult for?
   1. Myself
   2. A child under 14 years
   3. Someone older than 14 years (e.g. another family member)
3. Overall, how would you rate your satisfaction with the telephone consult?
   1. Very satisfied
   2. Satisfied
   3. Neither satisfied or dissatisfied
   4. Dissatisfied
   5. Very dissatisfied
4. What did you like about the telephone consult? [open ended]
5. What did you **not** like about the telephone consult? [open ended]
6. During the telephone consult, did your doctor or nurse listen to what you had to say:
   1. Yes, just as well or better than a face-to-face visit
   2. Yes, but not as well as in a face-to-face visit
   3. No
7. During the telephone consult, did your doctor or nurse spend enough time with you:
   1. Yes, just as well as or better than a face-to-face visit
   2. Yes, but not as well as in a face-to-face visit
   3. No
8. During the telephone consult, did your doctor or nurse treat you with kindness and understanding:
   1. Yes, just as well or better than a face-to-face visit
   2. Yes, but not as well as in a face-to-face visit
   3. No
9. During the telephone consult, did your doctor or nurse explain things to you in a way that

was easy to understand:

- 1. Yes, just as well or better than a face-to-face visit
  2. Yes, but not as well as in a face-to-face visit
  3. No

1. During the telephone consult, how concerned were you that the doctor or nurse could

not physically examine you?

- 1. Not at all concerned
  2. Slightly concerned
  3. Moderately concerned
  4. Very concerned
  5. Extremely concerned

1. How much did the telephone consult cost you?
   1. No cost
   2. Paid for by ACC (injury or accident)
   3. Less than a face-to-face visit
   4. The same as a face-to-face visit
   5. More than a face-to-face visit
   6. Don’t know
2. Have you ever had a telephone consult at a GP clinic/health centre **before** 23 March 2020

(i.e. before the coronavirus pandemic)?

- 1. Yes
  2. No

1. Since 23 March 2020, have you had a **virtual/video consult** with the GP clinic/health

Centre (e.g. using Skype or Zoom)?

- 1. Yes
  2. No

**If 20a, go to question 21, if 20b go to question 33**

1. Who was the virtual/video consult with?
   1. A doctor
   2. A nurse
   3. Someone else (please specify)
2. Who was the virtual/video consult for?
   1. Myself
   2. A child under 14 years
   3. Someone older than 14 years (e.g. another family member)
3. Overall, how would you rate your satisfaction with the virtual/video consult?
   1. Very satisfied
   2. Satisfied
   3. Neither satisfied or dissatisfied
   4. Dissatisfied
   5. Very dissatisfied
4. What did you like about the virtual/video consult? [open ended]
5. What did you **not** like about the virtual/video consult? [open ended]
6. During the virtual/video consult, did your doctor or nurse listen to what you had to say:
   1. Yes, just as well or better than a face-to-face visit
   2. Yes, but not as well as in a face-to-face visit
   3. No
7. During virtual/video consult, did your doctor or nurse spend enough time with you:
   1. Yes, just as well or better than a face-to-face visit
   2. Yes, but not as well as in a face-to-face visit
   3. No
8. During virtual/video consult, did your doctor or nurse treat you with kindness and

understanding:

- 1. Yes, just as well or better than a face-to-face visit
  2. Yes, but not as well as in a face-to-face visit
  3. No

1. During virtual/video consult, did your doctor or nurse explain things to you in a way

that was easy to understand:

- 1. Yes, just as well or better than a face-to-face visit
  2. Yes, but not as well as in a face-to-face visit
  3. No

1. During the virtual/video consult, how concerned were you that the doctor or nurse

could not physically examine you?

- 1. Not at all concerned
  2. Slightly concerned
  3. Moderately concerned
  4. Very concerned
  5. Extremely concerned

1. How much did the virtual/video consult cost you?
   1. No cost
   2. Paid for by ACC (injury or accident)
   3. Less than a face-to-face visit
   4. The same as a face-to-face visit
   5. More than a face-to-face visit
   6. Don’t know
2. Have you had a virtual/video consult at a GP clinic/health centre **before** 23 March 2020

(i.e. before the coronavirus pandemic)?

- 1. Yes
  2. No

1. Since 23 March 2020, have you had a **face-to-face visit** at the GP clinic/health centre?

(If you had more than one, please think about the most recent visit.)

- 1. Yes
  2. No

**If 33a, go to question 34, if 33b go to question 43**

1. Who was the face-to-face visit with?
   1. A doctor
   2. A nurse
   3. Someone else (please specify)
2. Who was the telephone consult for?
   1. Myself
   2. A child under 14 years
   3. Someone older than 14 years (e.g. another family member)
3. Overall, how would you rate your satisfaction with the face-to-face visit?
   1. Very satisfied
   2. Satisfied
   3. Neither satisfied or dissatisfied
   4. Dissatisfied
   5. Very dissatisfied
4. What did you like about the face-to-face visit? [open ended]
5. What did you **not** like about the face-to-face visit? [open ended]
6. During the face-to-face visit, did your doctor or nurse listen to what you had to say:
   1. Yes
   2. No
7. During the face-to-face visit, did your doctor or nurse spend enough time with you:
   1. Yes
   2. No
8. During the face-to-face visit, did your doctor or nurse treat you with kindness and

understanding:

- 1. Yes
  2. No

1. During the face-to-face visit, did your doctor or nurse explain things to you in a way

that was easy to understand:

- 1. Yes
  2. No

1. Since 23 March 2020, have you **got a prescription** from the GP clinic/health centre?
   1. Yes
   2. No

**If 43a, go to question 44, if 43b go to question 46**

1. How did you get the prescription?
   1. Ordered it online (through a portal such as Manage My Health, MyIndici)
   2. Phoned and left a message
   3. Phoned and spoke someone at the clinic
   4. Picked it up from the clinic
   5. Got it from a consult with the doctor or nurse
   6. Other (please specify)
2. How did you get the prescription **medicine**(s)?
   1. I (or someone on my behalf) took the prescription to a pharmacy and collected the medicine(s)
   2. The clinic sent the prescription to a pharmacy and I (or someone on my behalf) collected the medicine(s)
   3. The clinic sent the prescription to a pharmacy and the pharmacy sent the medicine(s) to me
   4. Other (please specify)

|  | 1. Were you aware of any the following services at the GP clinic **before** 23 March 2020 (before the coronavirus pandemic)? (you might not have used the service. if you don’t know, that’s fine) | 1. Were you aware of any the following services at the GP clinic **after** 23 March 2020 (during the coronavirus pandemic)? (you might not have used the service. if you don’t know, that’s fine) | 1. Which of the following services would you like your GP clinic/health centre to offer **in the future** (once the coronavirus pandemic is over)? |
| --- | --- | --- | --- |
| 1. Telephone consult with a doctor or nurse | (Y/N/DK) | (Y/N/DK) | (Y/N/DK) |
| 1. Virtual/video consult with a doctor or nurse | (Y/N/DK) | (Y/N/DK) | (Y/N/DK) |
| 1. Ordering prescriptions online | (Y/N/DK) | (Y/N/DK) | (Y/N/DK) |
| 1. Having prescriptions faxed to your pharmacist (so you don’t need to go into the clinic to pick up the prescription) | (Y/N/DK) | (Y/N/DK) | (Y/N/DK) |
| 1. Making appointments online | (Y/N/DK) | (Y/N/DK) | (Y/N/DK) |
| 1. Telephone triage, where you call the clinic and a nurse or doctor quickly calls you back to see what you need | (Y/N/DK) | (Y/N/DK) | (Y/N/DK) |
| 1. Same-day appointments for urgent problems | (Y/N/DK) | (Y/N/DK) | (Y/N/DK) |
| 1. Access to your notes through a secure online portal | (Y/N/DK) | (Y/N/DK) | (Y/N/DK) |
| 1. Access to your test results (e.g. blood tests, xrays) through a secure online portal | (Y/N/DK) | (Y/N/DK) | (Y/N/DK) |

1. Have you had contact with any of these **other** health care services since 23 March 2020 (when Alert Level 3 started)? [tick all that apply]
   1. No contact
   2. A community coronavirus testing centre
   3. An after-hours clinic (not an Emergency Department at a public hospital)
   4. Emergency Department at a public hospital
   5. Hospital inpatient admission
   6. Hospital outpatient clinic
   7. Pharmacy
   8. Healthline (0800 611 116 or 0800 358 5453 or text 1737)
   9. Other (please specify)
2. How concerned or not are you about How concerned or not are you about the impact of the coronavirus pandemic on your

health?

- 1. Not at all concerned
  2. Slightly concerned
  3. Moderately concerned
  4. Very concerned
  5. Extremely concerned

1. During the lockdown have you delayed seeking health care because of any of the following (tick all that apply):
   1. I have not delayed seeking health care
   2. Cost
   3. Lack of transport
   4. Fear of being infected with coronavirus
   5. Fear of infecting others with coronavirus
   6. Concern that health care workers are busy
   7. The health care I want isn’t available
   8. The health service postponed, rescheduled or delayed (e.g. an appointment, test, scan, surgery)
   9. Something else (please specify)

**If 51a, go to 52, otherwise go to 53**

52) How likely are you to delay seeking health care while in lockdown?

- 1. Extremely likely
  2. Very likely
  3. Moderately likely
  4. Slightly likely
  5. Not at all likely

1. Do you have any health conditions that mean you have to contact a GP clinic/health

centre on an on-going basis? (e.g. anxiety, arthritis, asthma, cancer, chronic respiratory disease, chronic pain, depression, diabetes, gout, heart disease, high blood pressure, stroke, etc)

- 1. Yes
  2. No

1. Do you think of yourself as disabled (or as having a disability)?
   1. Yes
   2. No
2. Has the coronavirus pandemic made it easier or more difficult to manage your health?
   1. Much easier
   2. A little easier
   3. Neither easier or more difficult
   4. A little more difficult
   5. Much more difficult
3. Is there anything else you would like to tell us about your experiences of health care

in the community during the pandemic? [open ended]

## Demographics

**Finally we have a few questions about yourself to help us better understand your answers**

1. In general, would you say that your health is:
   1. Excellent
   2. Very good
   3. Good
   4. Fair
   5. Poor
2. What is your gender?
   1. Male
   2. Female
   3. Gender diverse
   4. Prefer not to say
3. Which age group do you belong to:
   1. Under 18
   2. 18-24
   3. 25-34
   4. 35-44
   5. 45-54
   6. 55-64
   7. 65-74
   8. 75-84
   9. 85 or older
4. Which ethnic group or groups do you belong to (please tick all that apply):
   1. New Zealand European
   2. Māori
   3. Samoan
   4. Cook Islands Māori
   5. Tongan
   6. Niuean
   7. Chinese
   8. Indian
   9. Another ethnic group such as Dutch, Japanese or Tokelauan? Please specify
5. Which of these statements best describes your **current** work situation (during the lockdown):
   1. In paid employment (including self-employment) as before coronavirus
   2. In paid employment (including self-employment) but with reduced pay due to coronavirus
   3. In employment but not being paid because of coronavirus
   4. Not in paid employment and not looking for a job (for any reason, such as being retired, a caregiver, a homemaker, a full-time student)
   5. Unemployed (not due to coronavirus) and looking for a job (now or after lockdown)
   6. Unemployed as a direct result of coronavirus and looking for a job (now or after lockdown)
   7. Other (please specify)
6. How much do you agree or disagree with the following statement: Over the past 7 days,

my household has struggled to pay for basic living costs, such as food or accommodation.

- 1. Strongly agree
  2. Agree
  3. Neither agree nor disagree
  4. Disagree
  5. Strongly disagree

1. What is the postcode where you are currently living? (open ended)
2. Are you a New Zealand citizen or resident?
   1. Yes
   2. No
3. Do you have any feedback about this survey? [open ended]

## Follow up and contact details

Please note that any contact details you provide in this section will never be linked to your survey answers. All of your survey answers are anonymous.

**Copy of results**

1. Would you like to be sent a copy of the results of this survey?
   1. No thanks
   2. Yes please email or post me a copy of the results to [text box for email or postal address]

**Contact request**

1. We are looking for people who would be happy to be interviewed (by telephone or virtually, e.g. through Zoom or Skype) about their experiences of health care from GP clinics/health centres during the pandemic. For more information about this, see here [link to information sheet] or contact Fiona Imlach at [Fiona.imlach@vuw.ac.nz](mailto:Fiona.imlach@vuw.ac.nz) or 022 563 6565.

If you are happy for a researcher to contact you about an interview, please tell us your contact details. By giving us your details, you are not committed to take part in any further research, it just means we can contact you to ask.

Name:

Email address:

Phone number:

**Thank you very much for your time and for your feedback.**

**Please forward on the survey (add in link) to any of your contacts who would be interested in it.**

**If you have any questions about this survey, you can contact Fiona Imlach at** [**Fiona.imlach@vuw.ac.nz**](mailto:Fiona.imlach@vuw.ac.nz)

**If you have any questions or concerns about your health, please contact your GP clinic or call Healthline free on 0800 611 116.**

**For questions about coronavirus, please call 0800 358 5453 or visit** [**https://covid19.govt.nz/**](https://covid19.govt.nz/)
