# Supplementary material for: Telehealth consultations in general practice during a pandemic lockdown: survey and interviews on patient experiences and preferences
Source: BMC Fam Pract. 2020 Dec 13;21:269. doi: 10.1186/s12875-020-01336-1 (PMC7733693; doi:10.1186/s12875-020-01336-1)
Supplement: Supplementary file 2 — Additional file 2. Survey data comparison. [file 12875_2020_1336_MOESM2_ESM.docx]

# Additional file 2: Survey data comparison

To check the reliability of the data, we compared our sample to the New Zealand Health Survey 2018/19 (NZHS 2018/19)(1) which is a nationally representative survey of the normally resident population aged 15 years and older run by the Ministry of Health from July 2018 to June 2019. The NZHS 2018/19 asks respondents about their health status and health service use. A subset of respondents from the NZHS 2018/19 who were aged 18 years or older and who reported seeing a GP or a nurse about their own health within the past month were compared to those in our survey who had a telephone, video or in-person consult for themselves or someone else. Thus, our survey sample differed from the NZHS 2018/19 survey in that it included responses from people who could have contacted a practice about another person’s health, but the comparison provides a benchmark for the population of individuals attending general practice in usual circumstances.

Table 1 compares characteristics of the two survey samples. Both surveys had higher proportions of females, but this was larger in our survey, possibly because more women may have accessed care for others. Our selected sample had more people who were New Zealand European/Other and fewer Asian and Pacific peoples than the NZHS 2018/19. Our selected sample also had more people of middle age (45-54 years) and fewer older people (65+ years) compared to the NZHS 2018/19. The absence of older people could be because they did not see the survey advertisements through online networks or were not comfortable filling in an online survey. Our selected sample generally considered themselves in better health than those from the NZHS 2018/19.

Table 1: Comparison of survey respondents who had a telehealth or in-person consult (for themselves or someone else) to respondents from the NZHS 2018/19 who visited a GP or nurse about their own health in the last month

| **Characteristic** | **Survey respondents**  **n=690**  **%** | **NZHS 2018/19**  **n=3,902**  **% (95% confidence interval)** |
| --- | --- | --- |
| **Age group (years)** |  |  |
| 18-34 | 22.7 | 23.4 (21.7, 25.1) |
| 35-44 | 20.8 | 12.7 (11.5, 14.0) |
| 45-54 | 26.3 | 16.8 (15.3, 18.3) |
| 55-64 | 16.8 | 17.9 (16.4, 19.6) |
| 65+ | 13.5 | 29.2 (27.5, 31.0) |
| **Gender** |  |  |
| Female | 86.4 | 58.0 (56.1, 59.9) |
| Male | 12.2 | 42.0 (40.1, 43.9) |
| Other^1,2^ | 1.3 | - |
| **Prioritised ethnicity (in priority order)** |  |  |
| Māori | 10.1 | 12.4 (11.5, 13.3) |
| Pacific peoples | 1.3 | 5.4 (4.6, 6.3) |
| Asian | 3.0 | 9.3 (8.2, 10.5) |
| New Zealand European/Other | 85.5 | 73.0 (71.2, 74.7) |
| **Self-rated health** |  |  |
| Excellent | 11.6 | 9.8 (8.6, 11.1) |
| Very good | 37.3 | 32.0 (29.9, 34.1) |
| Good | 32.4 | 37.4 (35.4, 39.4) |
| Fair | 15.6 | 15.6 (14.0, 17.3) |
| Poor | 3.1 | 5.3 (4.5, 6.3) |
| ^1^ Those who answered ‘gender diverse’ or ‘prefer not to say’ were grouped together because of small numbers; ^2^ Only two options were available in the NZHS | | |

## References

1. Ministry of Health. Methodology report: New Zealand Health Survey 2018/19. Wellington: Ministry of Health; 2019.
